# An N-terminal deletion variant of HCN1 in the epileptic WAG/Rij strain modulates HCN current densities

**DOI:** 10.3389/fnmol.2015.00063

**Published:** 2015-11-03

**Authors:** Konstantin Wemhöner, Tatyana Kanyshkova, Nicole Silbernagel, Juncal Fernandez-Orth, Stefan Bittner, Aytug K. Kiper, Susanne Rinné, Michael F. Netter, Sven G. Meuth, Thomas Budde, Niels Decher

**Affiliations:** ^1^Institute for Physiology and Pathophysiology, Vegetative Physiology, Philipps-University of MarburgMarburg, Germany; ^2^Institute for Physiology I, Westfälische Wilhelms-UniversitätMünster, Germany; ^3^Department of Neurology, Westfälische Wilhelms-UniversitätMünster, Germany; ^4^Department of Neurology, University Medical Center, Johannes Gutenberg-University MainzMainz, Germany

**Keywords:** HCN, *I*_h_, thalamocortical relay neurons, absence epilepsy, WAG/Rij rat

## Abstract

Rats of the Wistar Albino Glaxo/Rij (WAG/Rij) strain show symptoms resembling human absence epilepsy. Thalamocortical neurons of WAG/Rij rats are characterized by an increased HCN1 expression, a negative shift in *I*_h_ activation curve, and an altered responsiveness of *I*_h_ to cAMP. We cloned HCN1 channels from rat thalamic cDNA libraries of the WAG/Rij strain and found an N-terminal deletion of 37 amino acids. In addition, WAG-HCN1 has a stretch of six amino acids, directly following the deletion, where the wild-type sequence (GNSVCF) is changed to a polyserine motif. These alterations were found solely in thalamus mRNA but not in genomic DNA. The truncated WAG-HCN1 was detected late postnatal in WAG/Rij rats and was not passed on to rats obtained from pairing WAG/Rij and non-epileptic August Copenhagen Irish rats. Heterologous expression in *Xenopus* oocytes revealed 2.2-fold increased current amplitude of WAG-HCN1 compared to rat HCN1. While WAG-HCN1 channels did not have altered current kinetics or changed regulation by protein kinases, fluorescence imaging revealed a faster and more pronounced surface expression of WAG-HCN1. Using co-expression experiments, we found that WAG-HCN1 channels suppress heteromeric HCN2 and HCN4 currents. Moreover, heteromeric channels of WAG-HCN1 with HCN2 have a reduced cAMP sensitivity. Functional studies revealed that the gain-of-function of WAG-HCN1 is not caused by the N-terminal deletion alone, thus requiring a change of the N-terminal GNSVCF motif. Our findings may help to explain previous observations in neurons of the WAG/Rij strain and indicate that WAG-HCN1 may contribute to the genesis of absence seizures in WAG/Rij rats.

## Introduction

Alterations in HCN channel expression and the corresponding pacemaker current *I*_h_ were attributed to pathological activity in the thalamocortical (TC) system, like SWD which are typical for childhood absence epilepsy (Budde et al., 2005; Kuisle et al., 2006; Biel et al., 2009; Kanyshkova et al., 2012). Several animal models were used to determine concepts of SWD generation (Crunelli and Leresche, 2002; Coenen and van Luijtelaar, 2003; Pinault and O’Brien, 2005; van Luijtelaar and Sitnikova, 2006; Huguenard and McCormick, 2007; Leresche et al., 2012). In WAG/Rij, SWD seem to start from the SSC, quickly spreading over the cortex, and invade the thalamus which provides a resonance circuitry for the amplification, spreading and entrainment (van Luijtelaar et al., 2011a,b; Lüttjohann and van Luijtelaar, 2015).

In WAG/Rij rats, systemic injections of *I*_h_ blockers resulted in a dose-dependent decrease in SWD pointing to a crucial role of HCN channels (van Luijtelaar et al., 2011b; van Luijtelaar and Zobeiri, 2014). Especially changed functionality of HCN1 channels with different characteristics in thalamus and cortex is involved in the increased seizure susceptibility (Ludwig et al., 2003; Strauss et al., 2004; Budde et al., 2005; Schridde et al., 2006; Kole et al., 2007; Kanyshkova et al., 2012). In the cortex, a reduction of HCN1 expression increases somato-dendritic excitability of pyramidal neurons leading to pathologically synchronized membrane currents and in turn to seizures (Strauss et al., 2004; Kole et al., 2007; Kanyshkova et al., 2012). Pharmacological treatment that prevents SWD also prevents the down-regulation of HCN1 in cortical areas of WAG/Rij rats (Blumenfeld et al., 2008). In the thalamus, the expression of HCN1 was higher in WAG/Rij rats than in non-epileptic rat strains (Budde et al., 2005; Kanyshkova et al., 2012). This change was accompanied by an increased *I*_h_ current density, a negative shift of the activation curve of *I*_h_, and a decreased sensitivity of *I*_h_ to cAMP in TC neurons which might result in an impairment to control the shift from burst to tonic firing. Furthermore an increase in thalamic expression of TRIP8b, an HCN channel ancillary subunit, was found (Lewis et al., 2009; Santoro et al., 2009; Zolles et al., 2009), coinciding with the more negative voltage dependence of *I*_h_ activation present in WAG/Rij TC neurons. In order to find possible evidence for specific changes in the HCN1 channel protein and an explanation for the opposite direction of altered HCN1 expression in thalamus versus cortex of the WAG/Rij strain, we cloned HCN1 from the thalamus of WAG/Rij rats. Here, we identified a HCN1 variant with an N-terminal deletion in the WAG/Rij rat strain which we named WAG-HCN1. We have functionally characterized WAG-HCN1 by heterologous expression and revealed a possible contribution of this HCN1 variant to the generation of absence epilepsy present in the WAG/Rij strain.

## Materials and Methods

### Full-length HCN1 Cloning

Total RNA was prepared from freshly dissected tissue by extraction with Trizol reagent according to the instructions of the manufacturer (RNeasy Lipid Tissue, Qiagen). Integrated QIAzol and RNeasy technologies of this Kit efficiently remove most of the DNA without DNase treatment. However, we used additional DNAseI digestions to remove also very small amounts of DNA. First-strand cDNA was primed with random hexamer primers (Invitrogen Life Technologies) from 0.5 to 1 μg of RNA and synthesized using the SuperScript II enzyme (Invitrogen) at 42°C for 50 min. Full-length cDNA for rat HCN1 and WAG-HCN1 were obtained by reverse transcription-polymerase chain reaction (RT-PCR) from total thalamic RNA. Three WAG/Rij rats from different offspring were used with three different DNA Taq polymerases: HS DNA Taq polymerase (TAKARA), HF2 DNA Taq polymerase (Clontech) and Pfu Turbo DNA Taq polymerase (Stratagene). Primers were as follows: forward, TTG GCC TCA AGC CCC CGG CGA GTC T; reverse, TCA TAA ATT CGA AGC AAA ACG GGG TTT (NM_053375, nucleotides 15–2807). PCR products corresponding to the full-size rat HCN1 were purified by agarose gel electrophoresis and cloned into pGEM-TEasy vector. Following bacteria transformation, clones containing HCN1 and WAG-HCN1 constructs were observed from all three cDNA preparations. All cDNA constructs for both HCN1 and WAG-HCN1 were established by sequencing of at least 10 different full-length cDNA clones for each preparation. For live cell imaging rat HCN1 and WAG-HCN1 were subcloned in the pEGFP-N vector (Clontech).

### Preparation of Genomic DNA

Preparation of genomic DNA (gDNA) was carried out by transfering small tissue samples from rat tails in lysis reagent (DirectPCR Tail, Peqlab) containing proteinase K (0.25 mg/mL). Tissue was incubated for 4 h at 55°C in a rotating hybridization oven followed by 45 min at 85°C in a thermomixer and short centrifugation. Small samples of lysate (1–1.5 μL) were used as template for PCR.

### Reverse Transcription-polymerase Chain Reaction

First-strand cDNAs were prepared as described above. Normalization was carried out against an endogenous housekeeping gene transcript for ß-actin. PCR was performed in a 20 μl reaction mixture using 0.5 U *Taq* polymerase (Qiagen); mixture in all cases contained 1.5 mM MgCl_2_, 0.2 mM of each dNTP, and 20 pmol of each primer using following cycling protocol: 3 min at 94°C; 35 cycles (25 cycles in case of ß-actin): 30 s at 94°C, 1 min at T_ann_, 1 min at 72°C; with a final elongation for 7 min at 72°C. The following primers were used: ß-actin, forward, ATT TGG CAC CAC ACT TTC TAC AAT, reverse, CTG CTT GCT GAT CCA CAT CTG C (NM_031144, nucleotides 253–1080), T_ann_ was 54°C; HCN1, forward, GCC TCA AGC CCC CGG CGA GTC T, reverse, ACG ATC CGA AGT GCT CTG GCG GTC TTG TAA (NM_053375, nucleotides 18–811), T_ann_ = 65°C.

### Semi-quantitative RT-PCR

All measurements for the relative comparison of HCN1 and WAG-HCN1 expression levels were performed within the exponential phase of PCR amplification. The optimal number of cycles required for detection of products in the linear range of amplification was determined for each of the cDNA-primer pair combinations in preliminary experiments. The level of mRNAs from WAG/Rij, ACI, WAG/Rji x ACI, and ACI x WAG/Rji rat tissues was normalized to each other using the constitutively expressed housekeeping gene β-actin. Quantification of each gene was achieved by the densitometric analysis of PCR products followed by calculation of the expression difference determined as a ratio of the PCR product of HCN1 to the PCR product of WAG-HCN1 using ImageJ (NIH, Bethesda).

### Expression of HCN Channels in *Xenopus* oocytes

*Xenopus* oocytes were prepared as previously described (Streit et al., 2011). Briefly, isolated oocytes were stored at 18°C in ND96 recording solution containing in mM: NaCl 96, KCl 2, CaCl_2_ 1.8, MgCl_2_ 1, HEPES 5; pH 7.4 with NaOH, supplemented with Na-pyruvate (275 mg/l), theophylline (90 mg/l), and gentamicin (50 mg/l). WAG-HCN1 and rat HCN1 were subcloned in pSGEM, linearized with NheI and cRNA was made using T7 polymerase. mHCN2 and hHCN4 were subcloned in pBF1. The mHCN2 construct was linearized with Eco72I and hHCN4 cDNA was linearized with Ade1. For *in vitro* transcription of HCN2 and HCN4 SP6 polymerase was used. Stages IV and V oocytes were injected with 5 ng of rat HCN1 or WAG-HCN1 cRNA. For co-expression, we used 5 ng HCN1 cRNA plus either 10 ng mouse HCN2, 25 ng human HCN4 or 1.25 ng human Kv1.1 cRNA, synthesized using the mMESSAGE mMACHINE Kit (Ambion). Standard TEVC experiments were performed at room temperature (21–22°C) in ND96 recording solution 2 days after the cRNA injection. Microelectrodes were fabricated from glass capillary tubes and filled with 3 M KCl. Tip resistance was in the range of 0.3–1.0 MΩ. TEVC recordings were performed using a TurboTEC-10CD Amplifier (npi) with a Digidata 1200 A/D-converter (Axon Instruments). For data acquisition the software pCLAMP7 (Axon Instruments) was used and data were analyzed with ClampFit10 (Axon Instruments). As current amplitudes after injection of a specific amount of cRNA varies from batch to batch, the current change by WAG-HCN1 from a batch of oocytes/experiments was normalized to the wild-type current. The ‘relative current’ provides the average current change analyzed from several batches of oocytes. This analysis more accurately reflects the current change that is observed in each individual batch of experiment, as it eliminates the fluctuations in overall expression levels (batch variance of amplitudes).

### Drugs

H-89, staurosporine, bisindolylmaleimide (all Cell Signaling Technology) and genistein (Sigma-Aldrich) were prepared from stock solution stored in DMSO and diluted in ND96 prior to recording. DMSO concentration was kept below 0.1% of the final solution. 8-Br-cAMP (Biaffin GmbH & Co KG) was directly diluted in ND96 recording solution prior to measurements.

### Animal Experiments

All animal experiments were carried out in accordance with EU Directive 2010/63/EU for animal experiments. The protocol was approved by the local animal care committee of the Regierungspräsidium Gießen.

### Statistics

All currents have been quantified at a potential of steady-state activation (-130 mV), unless stated otherwise. Results are reported as mean ± SEM (n = number of cells). Statistical differences were evaluated using an unpaired Student’s *t*-test, unless stated otherwise. Significance was assumed for ^∗^*p* < 0.05; ^∗∗^*p* < 0.01; ^∗∗∗^*p* < 0.001 and “n.s.” indicates a non-significant change.

## Results

### Rats of the WAG/Rij Strain Express an N-terminal Deletion Variant of HCN1 (WAG-HCN1)

**FIGURE 1:**
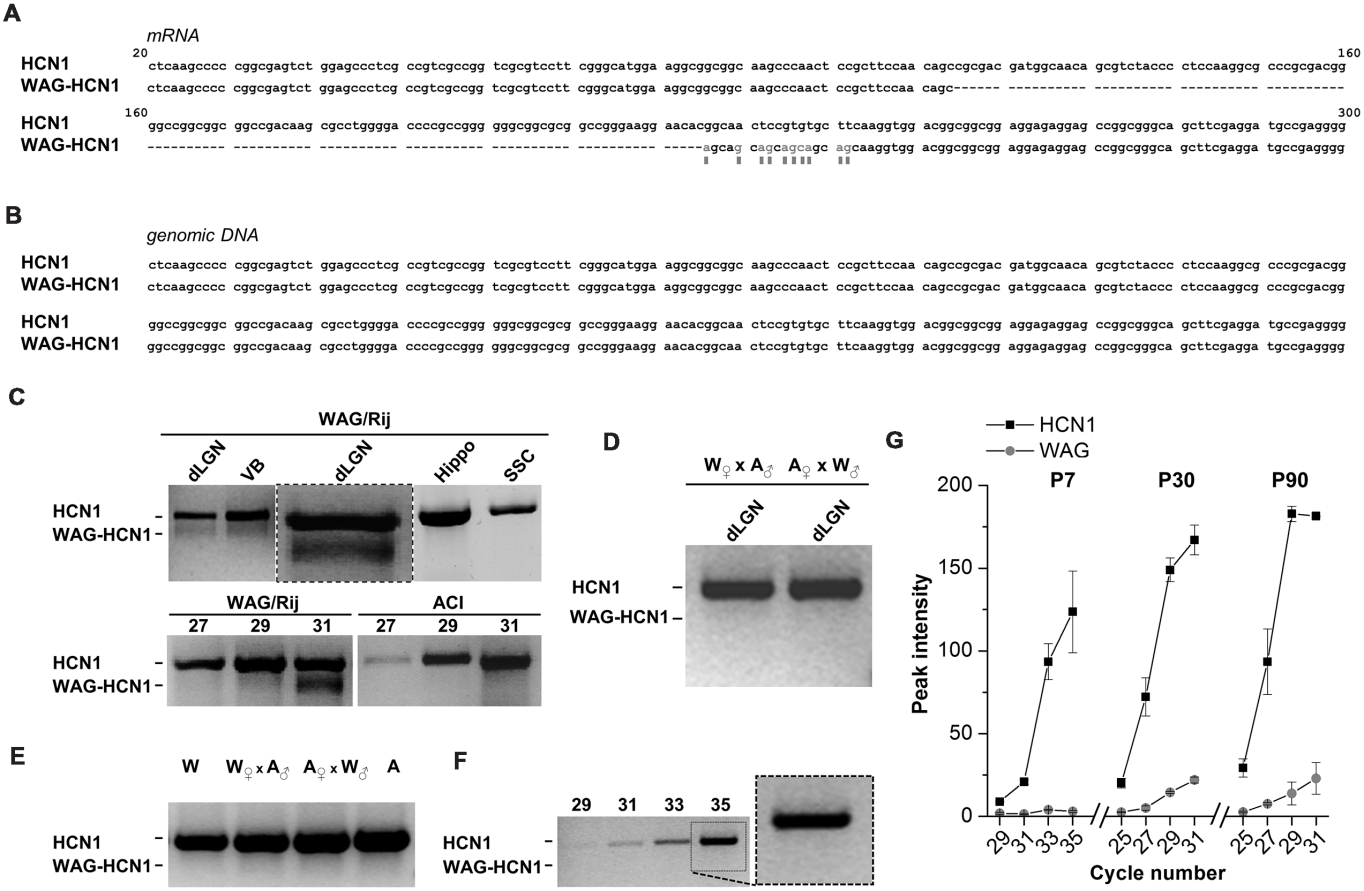
**Distribution of WAG-HCN1 channel in rat brain tissues. (A)** Alignment of the N-terminus of the mRNA sequences from rat HCN1 and WAG-HCN1. The WAG-HCN1 mRNA has a deletion of 111 nucleotides, and 10 nucleotides following the deletion are exchanged (gray letters and boxes). The sequences displayed are integral parts of exon 1 of rat HCN1. **(B)** Alignment of the genomic sequences corresponding to the N-terminal mRNA of rat HCN1 and WAG-HCN1 displayed in **(A)**. No differences were found (*n* = 5). **(C)** Upper panel, RT-PCR detection of HCN1 and WAG-HCN1 in different rat brain tissues: dLGN, ventrobasal thalamic complex (VB), hippocampus (Hippo), and S1 region of SSC. An additional PCR experiment of the dLGN with an expanded view of the double bands is illustrated in the middle (box). Lower panel, comparison of PCR detection of HCN1 and WAG-HCN1 (same experiment as the expanded view) in the LGN of WAG/Rij versus ACI rat after 27, 29, and 31 cycles. **(D)** RT-PCR detection of HCN1 using dLGN cDNA obtained from breaded WAG/Rij (W) and ACI (A) rats (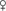 WAG/Rij x 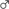 ACI and 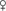 ACI x 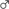 WAG/Rij, correspondingly). **(E)** PCR analysis of HCN1 and WAG-HCN1 isoforms in rat genomic DNA of WAG/Rij, ACl and their offspring. **(F,G)** Semi-quantitative RT-PCR analysis of HCN1 and WAG-HCN1 in the dLGN regions of WAG/Rij rats of different ages (P7, P30, P90). **(F)** Gel images of RT-PCR at P7. The numbers of PCR cycles are indicated. **(G)** Analysis of the peak intensities for PCR signals were plotted against cycle numbers for HCN1 and WAG-HCN1 at P7, P30 and P90. **(C–G)** The number of independent animals was *n* = 4 for all conditions.

Rats of the strain WAG/Rij typically show symptoms of absence-like epilepsy (van Luijtelaar and Sitnikova, 2006). Our aim was to analyze the contribution of HCN channel genes to this phenotype. While cloning of HCN1 channels from cDNA libraries obtained from thalamus of adult rats, we observed that the HCN1 channel from WAG/Rij rats harbors an N-terminal deletion (**Figure 1**). This deletion variant (WAG-HCN1) was only observed in thalamic cDNA libraries of epileptic rats (**Figure 1C**), but not in cDNA of the non-epileptic control strain ACI (**Figure 1C**, lower panel). An alignment with the *Rattus norvegicus* HCN1 mRNA (NM_053375) revealed a deletion of 111 bases and several base exchanges in the N-terminal region (**Figure 1A**). Sequencing of genomic DNA from the thalamus of WAG/Rij rats revealed that this deletion was not present on chromosome 2 when compared to the genomic sequence of *Rattus norvegicus* (NW_047620; **Figure 1B**). A PCR analysis using specific primers of the N-terminal region generated two bands with the expected length of 794 basepairs (HCN1) and 683 basepairs (WAG-HCN1) in samples from two thalamic areas (dLGN, VB) but not from hippocampus (Hippo) and primary SSC (**Figure 1C**, upper panel). In order to explore the hereditary of the WAG-HCN1 trait, we bred WAG/Rij with the corresponding non-epileptic strain (Depaulis and van Luijtelaar, 2006), the ACI rats (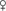 WAG/Rij x 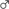 ACI; 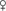 ACI x 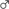 WAG/Rij). While the WAG-HCN1 PCR band was detected in all dLGN tissue samples from WAG/Rij rats (collected over many generations from 2005 to 2012), no positive PCR signals were found in WAG/Rij-ACI hybrids (two litters with a total of 16 pups were analyzed; **Figure 1D**). As for WAG/Rij and ACI rats, no PCR signals of the WAG-HCN1 variant were found in WAG/Rij-ACI hybrids using genomic DNA as template (**Figure 1E**). Next, we assessed the developmental expression level of WAG-HCN1 using cDNA from dLGN at P7, P30, and P90. Due to the high GC-content of the HCN1 N-terminus, we could not design primers suitable for a quantitative real time PCR analysis of the two variants. Thus, the expression levels were analyzed using a semi-quantitative PCR approach and peak densitometry of the gel bands (**Figures 1F,G**). While WAG-HCN1 was not detected at P7 (**Figures 1F,G**), the WAG-HCN1 variant was up-regulated at postnatal ages P30 and P90 (**Figure 1G**). Note that the relative amount of WAG-HCN1 mRNA identified here appeared very small, with only approximately 10% of the transcripts.

### The WAG-HCN1 Deletion Leads to Increased HCN1 Current Amplitudes (Gain-of-function) While Gating Behavior of the Channel Remains Unaltered

**FIGURE 2:**
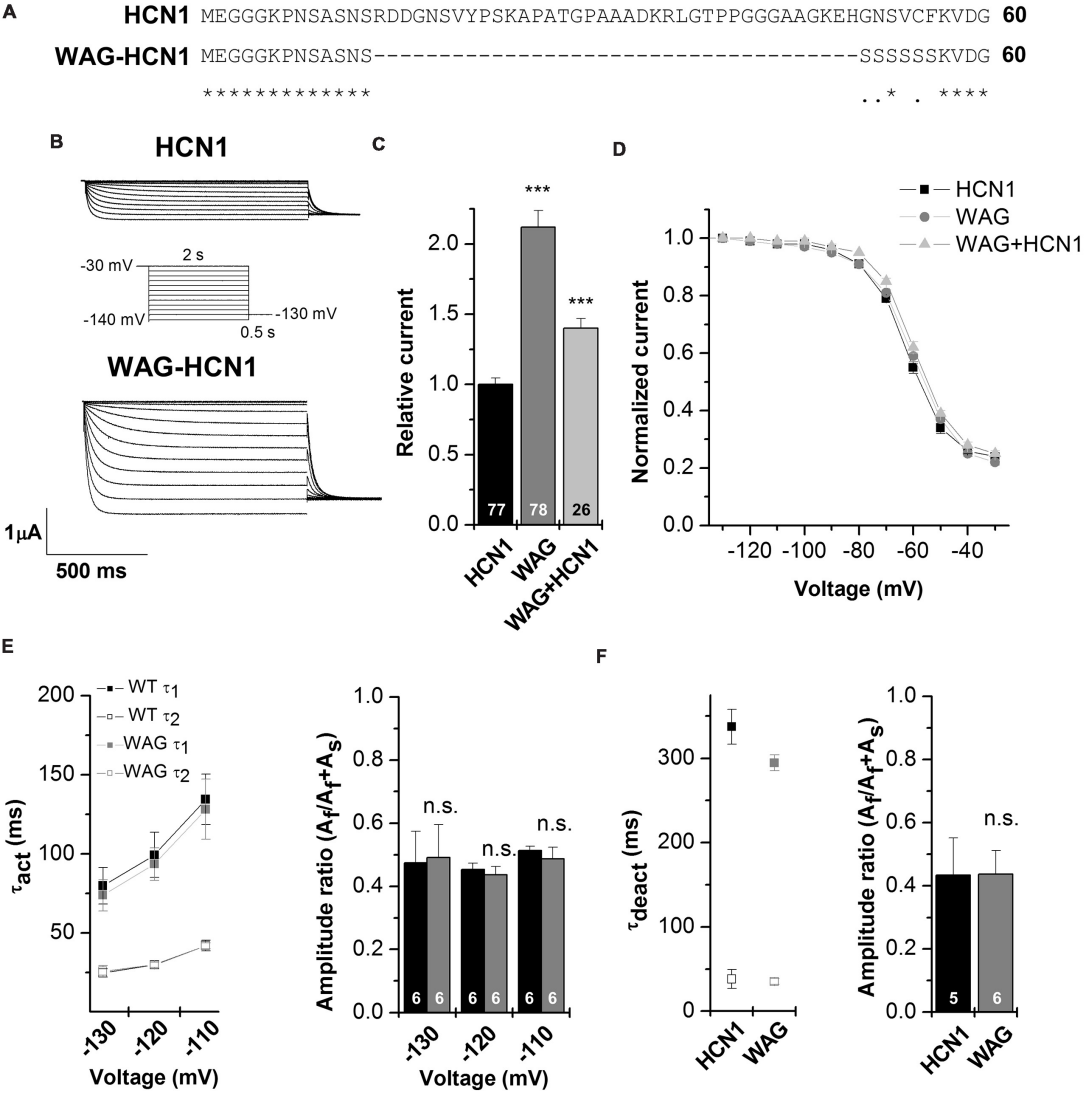
**Electrophysiological characterization of the WAG-HCN1 channel. (A)** Alignment of the first 60 amino acids of the N-terminus from rat HCN1 and WAG-HCN1. The WAG-HCN1 channels have a deletion of 37 amino acids and six amino acids directly following the deletion (GNSVCF) are exchanged to serine residues. **(B)** Representative measurements of HCN1 (top) and WAG-HCN1 (bottom) currents. Oocytes were held at -30 mV and voltage steps of 2 s ranging from -30 to -140 mV were applied, followed by a step to -130 mV to record tail currents, see the illustrated voltage protocol. **(C)** Comparison of current amplitude of HCN1 (black), WAG-HCN1 (gray) and heteromeric HCN1/WAG-HCN1 currents (light gray), analyzed at -130 mV. The number of experiments are indicated in the bar graphs. **(D)** Conductance-voltage relationships of HCN1 (black), WAG-HCN1 (gray) and heteromeric HCN1/WAG-HCN1 currents (light gray). **(E)** Slow and fast time constants of activation at three different potentials, analyzed for HCN1 (black) and WAG-HCN1 (gray) using a bi-exponential fit. Right panel illustrates the amplitude ratio of the fast component over the sum of the fast and slow component. The number of experiments are indicated in the bar graphs. **(F)** Slow and fast time constants of deactivation analyzed at +20 mV for HCN1 (black) and WAG-HCN1 (gray) using bi-exponential fits. Right panel illustrates the amplitude ratio of the fast component of deactivation over the sum of the fast and slow component. The number of experiments are indicated in the bar graphs. ^∗∗∗^*p* < 0.001.

**Figure 2A** illustrates an alignment of *Rattus norvegicus* HCN1 and WAG-HCN1 N-terminal protein sequences. The sequence of the WAG-HCN1 channel lacks 37 amino acids in the proximal N-terminus of the channel. In addition, WAG-HCN1 has a stretch of six amino acids, directly following the deletion, where the wild-type sequence GNSVCF is changed to a polyserine motif (**Figure 2A**). Next, we analyzed the functional differences caused by the altered N-terminus by electrophysiological recordings using the *Xenopus* oocyte expression system. Expression of WAG-HCN1 in oocytes yields the typical fast activating HCN1 currents (**Figure 2B**) upon hyperpolarization of the plasma membrane. Interestingly, the currents after injection of a WAG-HCN1 construct were larger than rat HCN1 currents (**Figures 2B,C**). After 48 h of expression, the WAG-HCN1 currents were by a factor of 2.19 ± 0.13 (*n* = 78) larger than rat HCN1 currents (1.00 ± 0.05, *n* = 77; **Figure 2C**). Co-expression of WAG-HCN1 with rat HCN1 also increased current amplitudes, albeit to a lesser extent (**Figure 2C**). As co-expression of WAG-HCN1 with rat HCN1 (*n* = 26) yielded currents with an intermediate amplitude of 1.40 ± 0.07 (*n* = 26; **Figure 2C**), we conclude that the increased current amplitude is not transduced to the heteromeric channel in a dominant-active manner. The voltage dependence of activation of rat HCN1, WAG-HCN1 and the heteromeric channels were not altered (**Figure 2D**). The voltage of half-maximal activation (V_1/2_) was -62.59 ± 0.49 mV (*n* = 37) for rat HCN1, -60.21 ± 0.69 mV (*n* = 35) for WAG-HCN1, and -58.69 ± 0.66 mV (*n* = 20) for the heteromeric channels. Next, we analyzed the activation (**Figure 2E**) and deactivation kinetics (**Figure 2F**) of WAG-HCN1 channels. Activation kinetics of HCN1 and WAG-HCN1 currents were analyzed in the voltage range of -110 to -130 mV, using bi-exponential fits (**Figure 2E**). The relative amplitudes of the fast and slow component of activation are shown in **Figure 2E** as a ratio of fast component over the sum of both components (right panel, *n* = 6 each). We found that the activation kinetics were similar for rat HCN1 and WAG-HCN1. Analyzing the kinetics of deactivation at +20 mV, using bi-exponential fits, we found that also the deactivation kinetic of WAG-HCN1 is not altered in comparison to rat HCN1 (**Figure 2F**). Summarizing, WAG-HCN1 channels produce about 2.2-fold more current than rat HCN1 channels, without any apparent changes in voltage dependence, as well as activation and deactivation kinetics. Preliminary fluorescence imaging experiments indicate that WAG-HCN1 channels have an increased surface expression, while Western blot experiments with COS7 cells transfected with either HCN1 or WAG-HCN1 revealed that the global protein expression of HCN1 and WAG-HCN1 were similar (data not shown). In summary, given also the unaltered current characteristics (gating properties) of WAG-HCN1, our data suggests an increased surface expression as a mechanism (**Figures 2D–F**) for the increased WAG-HCN1 current amplitudes (**Figure 2C**).

### Co-expression with WAG-HCN1 Reduces Current Amplitudes of Other HCN Family Members

**FIGURE 3:**
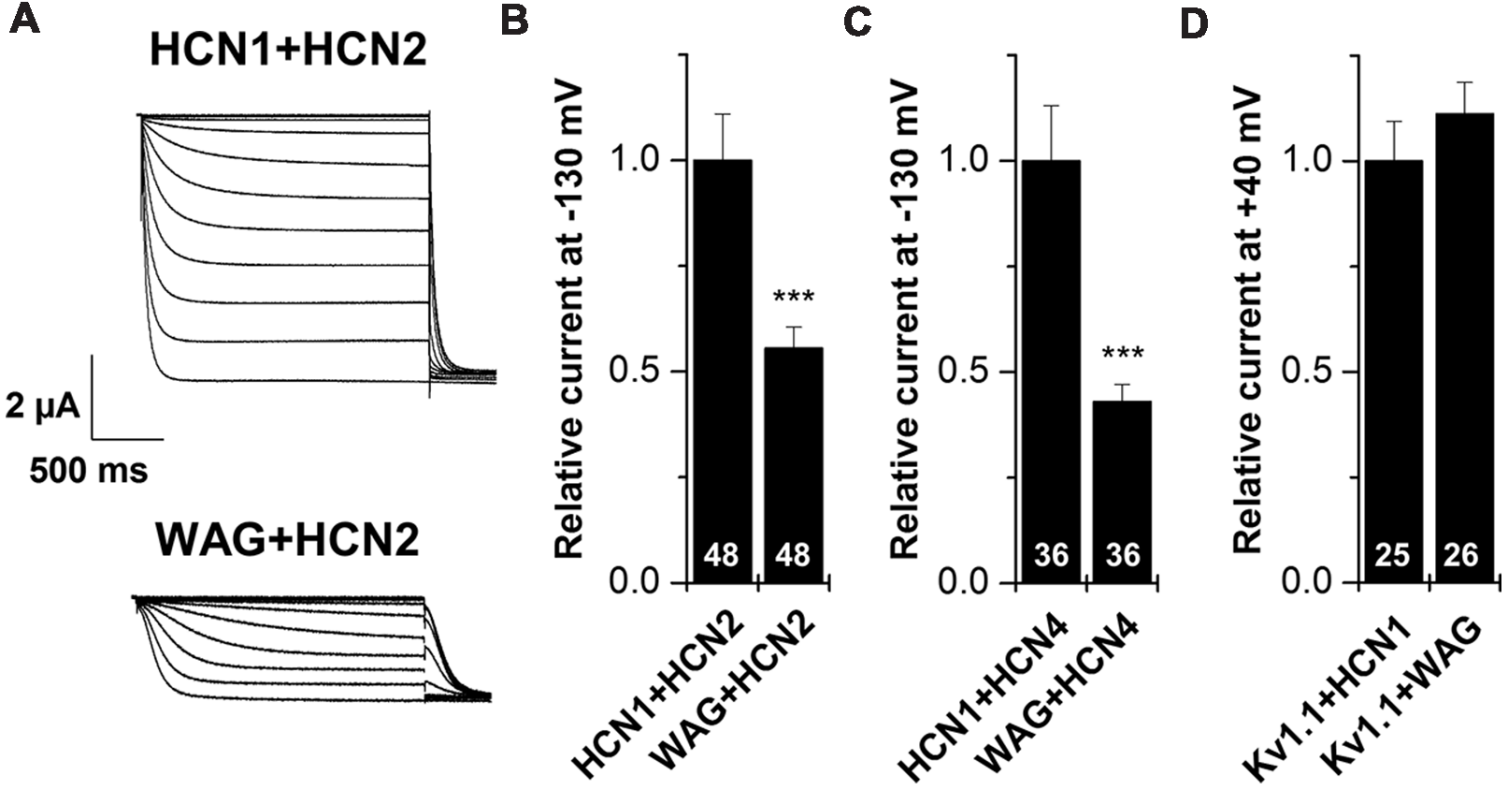
**Co-expression of HCN1 or WAG-HCN1 with other HCN family members. (A)** Representative current traces of the co-expression of HCN1 or WAG-HCN1 with HCN2. **(B)** Co-expression with WAG-HCN1 causes a significant decrease in current amplitude analyzed at -130 mV. The number of experiments are indicated in the bar graphs. **(C)** Co-expression of WAG-HCN1 with HCN4 shows a significant decrease in current amplitude analyzed at -130 mV. The number of experiments are indicated in the bar graphs. **(D)** Relative current amplitudes (at +40 mV) for Kv1.1 after co-expression with HCN1 or WAG-HCN1 with HCN2. The number of experiments are indicated in the bar graphs. ^∗∗∗^*p* < 0.001.

HCN1 channels are mainly expressed in the brain, where they can heteromerize with different HCN isoforms to form functional channels (Chen et al., 2001; Much et al., 2003). Therefore, we characterized the WAG-HCN1 variant also by co-expressions with other HCN family members, expressed in TC neurons. For co-expression, oocytes were injected with 5 ng of HCN1 cRNA together with 10 ng of HCN2 or 25 ng of HCN4 cRNA. The amounts of cRNAs were previously determined in order that every channel generates similar current amplitudes when injected alone (data not shown). The most interesting and somewhat unexpected observation was that co-expressions of WAG-HCN1 with either HCN2 or HCN4 yielded reduced current amplitudes of the heteromeric channels (**Figures 3A–C**). When WAG-HCN1 was co-expressed with HCN2, currents were reduced by 45% in comparison to the co-expression with rat HCN1 (*n* = 48; **Figures 3A,B**). Co-expression of WAG-HCN1 with HCN4 led to an even more pronounced reduction of current amplitudes. Heteromeric HCN1/HCN4 currents were reduced by 57% due to the presence of WAG-HCN1 (*n* = 36; **Figure 3C**). Note that the current reduction of HCN2 or HCN4 by WAG-HCN1 are most likely, due to the low expression levels of WAG-HCN1, less pronounced than observed in these experiments.

As a control, we have co-expressed Kv1.1 with either HCN1 or WAG-HCN1 and analyzed the Kv1.1 current amplitudes (**Figure 3D**). The fact that the Kv1.1 current amplitudes are not suppressed by co-expression with WAG-HCN1 further supports that the HCN2 or HCN4 current reduction are not caused by a limited protein translation efficiency due to the increased expression levels of WAG-HCN1. These findings indicate that WAG-HCN1 directly interacts with HCN2 or HCN4 to decrease currents encoded by the respective heteromeric channels.

### WAG-HCN1/HCN2 Heteromers have a Reduced cAMP Sensitivity

**FIGURE 4:**
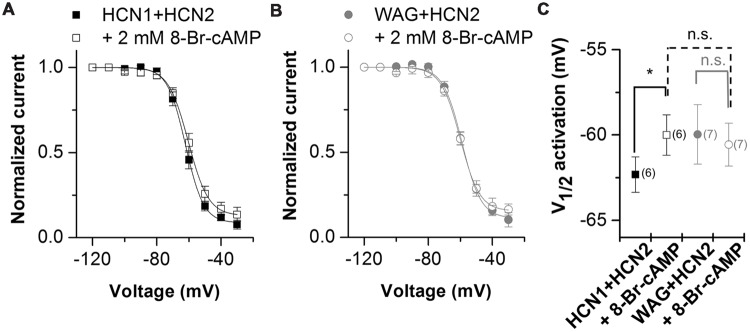
**Properties of heteromeric channels of WAG-HCN1 with HCN2. (A)** Conductance-voltage relationship of rat HCN1 co-expressed with HCN2 stored in theophyllin-free solutions, recorded before (black squares) and after 5 min of application of 2 mM 8-Br-cAMP (open squares). **(B)** Conductance-voltage relationship of WAG-HCN1 co-expressed with HCN2 stored in theophyllin-free solutions, recorded before (gray circles) and after 5 min of application of 2 mM 8-Br-cAMP (open circles). **(C)** V_1/2_ of activation for heteromeric channels of rat HCN1 or WAG-HCN1 co-expressed with HCN2. The number of experiments for **(A–C)** are indicated in parenthesis within the panel **(C)**. ^∗^*p* < 0.05.

Hyperpolarization-activated inward current from TC neurons has a strong contribution of HCN2 channels (Ludwig et al., 2003; Kanyshkova et al., 2009). As the *I*_h_ current in TC neurons of the rat WAG/Rij strain has an altered voltage dependence of activation and cAMP-responsiveness (Budde et al., 2005; Kanyshkova et al., 2012), we studied the heteromeric channels of WAG-HCN1 with HCN2 in more detail. As described above, for co-expression oocytes were co-injected with 5 ng HCN1 or WAG-HCN1 cRNA and 10 ng HCN2 cRNA. Current-voltage relationship of heteromeric channels of HCN2 together with WAG-HCN1 was not significantly altered (**Figures 4A–C**). While channels from a co-expression of HCN2 with HCN1 had a V_1/2_ of activation of -62.3 ± 1.0 mV (*n* = 6), heteromeric channels with WAG-HCN1 had a V_1/2_ of -60.0 ± 1.7 mV (*n* = 7; **Figures 4A–C**). Next we aimed to analyze whether there was an altered cAMP-responsiveness of heteromeric WAG-HCN1/HCN2 channels. In general, the activation curve of HCN1 is more depolarized than for the other HCN family members and additional application of cAMP affects the activation curve only marginally. The reason for this effect is that at typical basal concentrations of cAMP in neurons, HCN1 channels are not inhibited by the C-linker/CNBD region and already reveal a right shifted position of the activation curve (Lolicato et al., 2011; Chow et al., 2012). Therefore, for the recordings with 8-Br-cAMP, theophylline was omitted from the storage solution, to have the lowest possible cAMP levels. Measurements were made before and after 5 min of incubation with 2 mM 8-Br-cAMP. Application of 2 mM 8-Br-cAMP significantly shifted the activation of heteromeric HCN1/HCN2 channels (ΔV_1/2_) by +2.3 mV (*n* = 6; **Figures 4A,C**). The rather small effect of 8-Br-cAMP on HCN1/HCN2 channels in the oocyte expression system might be due to the still high cAMP levels present in whole cell oocytes which may be already close to the maximally possible cAMP shift. Thus, only small additional shifts can be achieved by 8-Br-cAMP. Similar small cAMP responses in whole cell oocytes were observed for homomeric HCN1 and HCN2 channels alone (data not shown). Most importantly, 2 mM 8-Br-cAMP did not cause a significant shift in the V_1/2_ of heteromeric WAG-HCN1/HCN2 channels (ΔV_1/2_ = -0.6 mV; *n* = 7; **Figures 4B,C**).

### The Polyserine Motif Present in the WAG-HCN1 N-terminus does not Cause the HCN Current Gain-of-function

**FIGURE 5:**
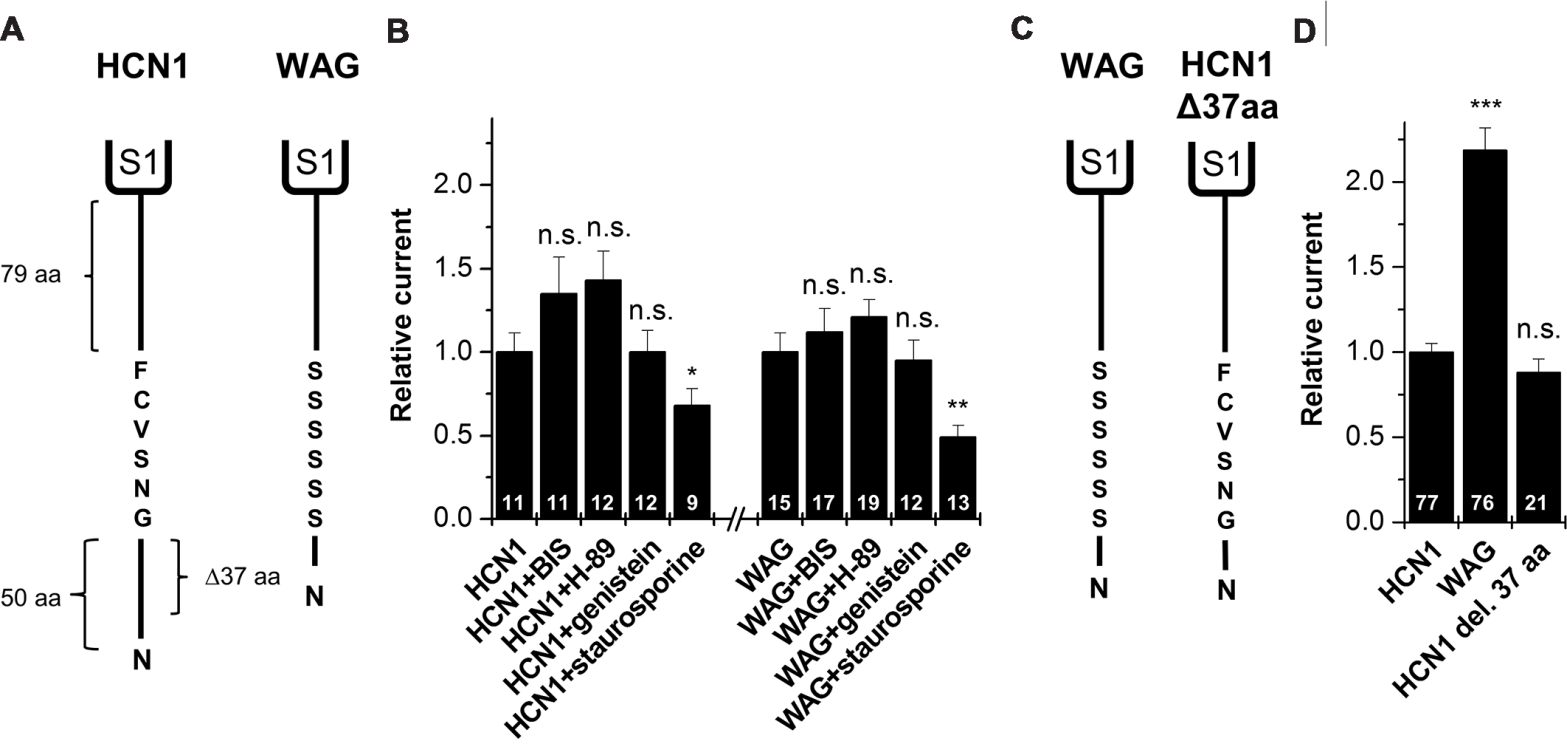
**Molecular correlate for the WAG-HCN1 gain-of-function. (A)** Cartoon depicting the N-termini of rat HCN1 (left) and WAG-HCN1 (right). The N-terminus of rat HCN1 has a length of 135 amino acids. WAG-HCN1 has a deletion of 37 amino acids and a novel polyserine motif of six amino acids. **(B)** Measurement of current amplitude of HCN1 and WAG-HCN1 48 h after injection and incubation in ND96 storage solution, supplemented with different protein kinase inhibitors (1 μM bisindolylmaleimide (BIS), 1 μM H-89, 10 μM genistein, or 1 μM staurosporine). Currents were normalized to the HCN1 construct incubated in normal storage solution. The number of experiments are indicated in the bar graphs. **(C)** Comparison of WAG-HCN1 (left) with a mutated WAG-HCN1 (HCN1-Δ37-aa) where the polyserine motif has been mutated to the wild-type sequence GNSVCF (right). This construct harbors only the deletion and not the polyserine stretch. **(D)** Current amplitudes of HCN1, WAG-HCN1 and HCN1-Δ37-aa analyzed at -130 mV. HCN1-Δ37-aa has a current amplitude similar to that of rat HCN1 (0.88 ± 0.08). The number of experiments are indicated in the bar graphs. ^∗^*p* < 0.05; ^∗∗^*p* < 0.01; ^∗∗∗^*p* < 0.001.

As mentioned above, a comparison of the protein sequence of rat HCN1 channels with that of WAG-HCN1 shows that there are two major differences in the N-terminal segment of the HCN1 channel. First, there is a deletion of 37 amino acids in the proximal N-terminus of the WAG-HCN1 variant and second, there is a stretch of six amino acids following the deletion which is altered to a polyserine-motif (**Figure 5A**). An increased phosphorylation of channels at the polyserine motif might cause an increased surface trafficking or an altered gating. We checked for differences in the phosphorylation dependent modulation of HCN1 and WAG-HCN1 utilizing different protein kinase inhibitors. Bisindolylmaleimide I (1 μM) was used to inhibit protein kinase C (Davis et al., 1989) and H-89 (1 μM) to block cAMP dependent protein kinase A (Chijiwa et al., 1990). Genistein (10 μM) was tested as a blocker of tyrosine kinases (Akiyama et al., 1987). Finally, staurosporine (1 μM), which is also known to induce cell apoptosis (Couldwell et al., 1994; Yue et al., 1998), was used as an unspecific kinase inhibitor, affecting PKA, PKC, PKG, CAMKII and myosin light chain kinase (Ruegg and Burgess, 1989). Oocytes were injected with either HCN1 or WAG-HCN1 and stored for 48 h in ND96 solution in the absence or presence of the kinase blockers. HCN1 and WAG-HCN1 showed a similar response to the kinase blockers (**Figure 5B**). Both channels showed only minor changes in current amplitudes by blockers of the PKA, PKC, and tyrosine kinases. Staurosporine significantly reduced HCN1 and WAG-HCN1 (**Figure 5B**), while the current reduction was not significantly different for HCN1 and WAG-HCN1 (*p* = 0.14). We therefore conclude that the gain-of-function of WAG-HCN1 is not caused by an altered phosphorylation at the WAG-HCN1-specific polyserine-motif.

### Gain-of-function of WAG-HCN1 Requires the Replacement of the Native N-terminal GNSVCF Sequence

To assess whether the deletion of the 37 amino acids or the exchange of the subsequent GNSVCF sequence is responsible for the WAG-HCN1 gain-of-function, we constructed a WAG-HCN1 channel harboring only a deletion of 37 amino acids (HCN1-Δ37-aa), while the rat HCN1 sequence following the deletion (GNSVCF) was not changed to polyserine (**Figure 5C**). Next, we compared current amplitudes of HCN1, WAG-HCN1 and HCN1Δ37aa expressed in oocytes. TEVC recordings were performed 2 days after injection of 5 ng of the respective cRNAs (**Figure 5D**). While WAG-HCN1 showed a strong gain-of-function, the HCN1-Δ37-aa construct produced currents with similar amplitudes as rat HCN1 (**Figure 5D**). Thus, the gain-of-function of the WAG-HCN1 channel does not result from the 37 amino acid deletion alone, as it needs the amino acid exchanges in the GNSVCF sequence. We propose that the deletion together with the changes of this motif lead to a loss of retention of the channels, presumably due to an impaired interaction with another protein.

## Discussion

Based on mRNA analyses we here identified a variant of rat HCN1 channels, termed WAG-HCN1, which was only present in the thalamus of WAG/Rij rats ≥ P30. When expressed alone in oocytes or co-expressed with other HCN channel subtypes, WAG-HCN1 revealed a number of interesting properties. In comparison to normal HCN1 the WAG variant generated two-times more current and exerted also a positive effect when both variants were co-expressed. Co-expression with other HCN channels subtypes (HCN2, HCN4), but not Kv1.1 channels, resulted in reduced current amplitudes. Furthermore, the co-expression of HCN2 with WAG-HCN1, but not HCN1, abolished the modulation of the resulting current by externally applied 8-Br-cAMP. Although the protein expression of WAG-HCN1 channels in WAG/Rij rats was not ultimately proven here, the present findings may help to explain some aspects of the experimental observations made with the WAG/Rij strain. This assumption may indeed apply since early infantile epileptic syndromes and idiopathic generalized epilepsy has been associated with mutations in HCN1 and HCN2 channel genes (DiFrancesco and DiFrancesco, 2015).

Rats of the WAG/Rij strain serve as a well-established animal model for absence epilepsy and changes in HCN1 properties were previously proposed to contribute to the development of seizures (Strauss et al., 2004; Budde et al., 2005; Schridde et al., 2006; Kole et al., 2007; Kanyshkova et al., 2012). Several groups found changes in HCN1 properties, with a reduction or an increase of HCN1, depending on the animal model and tissue examined (**Table 1**). In the neocortex and hippocampus of the WAG/Rij strain HCN1 is reduced on protein level but not the mRNA level (Strauss et al., 2004). This reduction was proposed to lead to an increased excitability of neurons in the SSC which may facilitate the generation of SWDs and result in epileptic seizures (Strauss et al., 2004; Kole et al., 2007). The hypothesis that a parallel reduction of *I*_h_ in cortical and thalamic areas can lead to absence epilepsies, is supported by the fact that genetic and spontaneous HCN2 knock-out mouse develops spontaneous absence seizures (Ludwig et al., 2003; Chung et al., 2009). In contrast to data from the neocortex and hippocampus where HCN1 seems to be down-regulated, increased hyperpolarization-activated currents in the thalamocortical neurons are linked to the generation of seizures (Di Pasquale et al., 1997; Budde et al., 2005; Kuisle et al., 2006; Kanyshkova et al., 2012). We reported an increased mRNA and protein expression of HCN1 in thalamocortical neurons of the WAG/Rij strain compared to ACI rats, while the relative expression of other HCN family members were not altered (Kanyshkova et al., 2012). Stronger HCN1 expression correlated with an increased *I*_h_ current density in WAG/Rij rats. These differences were first detected between P15 and P30 and peaked at P90. The latter age correlated with the onset of absence epilepsies in these animals. These alterations in HCN1 were accompanied by a developmental up-regulation of HCN2 and HCN4 in WAG/Rij and ACI rats, but no differences between strains were found for the expression levels of these two isoforms. Summarizing, in TC neurons of the WAG/Rij strain the native *I*_h_ was characterized by an increased current density, a negative shift in the activation curve, and a decreased cAMP-sensitivity (Budde et al., 2005; Kanyshkova et al., 2012). There was more TRIP8b protein detectable in TC neurons of WAG/Rij versus ACI rats, which is in agreement with the more hyperpolarized activation curves and reduced cAMP-sensitivity of *I*_h_ in TC neurons of the WAG/Rij strain (Kanyshkova et al., 2012).

**Table 1:** Studies of *I*_h_ and HCN channels in thalamocortical relay neurons of rat (WAG/Rij; GAERS) and mouse (HCN2-/- knock-out) models of Absence epilepsy.

Reference	Animal	Tissue	Expression				*I*_h_			
			*HCN1*	*HCN2*	*HCN3*	*HCN4*	*V_1/2_*	*Amplitude*	*cAMP sensitivity*	*τ _act_*
Kanyshkova et al., 2012	WAG/Rij versus ACI	TC relay neurons (dLGN, P90; VB, P30)	**↑** Prot. (dLGN) / = Prot. (VB)	const.	(↑) Prot. on P7	const.	-7.9 mV (dLGN) -3 mV (VB)	**↑** ~1.5-fold (dLGN)	Reduced	Faster
Budde et al., 2005	WAG/Rij versus ACI	TC relay (dLGN, P16–29)	**↑** mRNA and Prot. (dLGN)	const.	const.	const.	-5.2 mV (dLGN) -2.8 mV (VB)	Not analyzed systematically	Reduced	Faster
Strauss et al., 2004	WAG/Rij versus ACI and Wistar	Pyramidal (layers 2–3) somatosensory cortex	**ccc** Prot.^∗^ / = mRNA	const.	const.	const.	+9 mV (somatic)	**↓** ~0.5-fold (somatic)	n.t.	Slower
Kole et al., 2007	WAG/Rij versus Wistar	Pyramidal (layer 5) somatosensory cortex	**↓** Prot. (68%)	const.	n.t.	n.t.	const. (dendritic)	**↓** ~0.5-fold (dendritic)	n.t.	n.t.
Ludwig et al., 2003	HCN2^-/-^ mouse	TC relay neurons and hippocampus	const.	-/-	const.	const.	-27 mV (TC) -2 mV (CA)	**↓** 80% (TC) **↓** 39% (CA)	Reduced	n.t.
Cain et al., 2014	GAERS versus NEC	TC relay neurons (VB, P7–P150)	**↑** Prot. (VB)	const.	**↑** Prot. (VB)	const.	const.	**↑** ~2-fold	n.t.	n.t.
Kuisle et al., 2006	GAERS versus NEC	TC relay neurons (VB, 19–24 days, 3–8 months)	**↑** mRNA (VB, NRT)	const.	n.t.	const.	const.	const.	Reduced	n.t.

*CA pyramidal neurons of the hippocampus; const. = constant; dLGN = dorsal lateral geniculate nucleus; GAERS = Genetic Absence Epilepsy in Rats from Strasbourg; NEC = non-epileptic controls; NRT = Nucleus reticularis thalami; n.t. = not tested; Prot. = Protein; TC = thalamocortical; VB = ventrobasal thalamic complex; ‘↑’ = up-regulated; ‘↓’ = down-regulation; ‘ = ’ = not regulated. ^∗^hippocampus ↓46%, neocortex ↓21%, cerebellum ↓57%.*

In addition, the WAG-HCN1 variant which we have identified here, may also help to explain further aspects of the experimental observations made with the WAG/Rij strain in different brain regions (**Table 1**): (1) Our electrophysiological studies revealed a gain-of-function for WAG-HCN1 without changes in channel kinetics. This data is in good agreement with an increased *I*_h_ current amplitude and faster activation kinetics of thalamocortical *I*_h_ currents of the WAG/Rij strain. (2) The WAG-HCN1 variant forms heteromers with HCN1, HCN2, and HCN4. While heteromeric channels with HCN1 have increased current amplitudes, WAG-HCN1 suppresses heteromeric currents with HCN2 and HCN4. The reduction of HCN2 and HCN4 which have slower activation kinetics than HCN1 might contribute to the faster activation kinetics observed in TC neurons of the WAG/Rij strain. (3) Heteromeric channels of WAG-HCN1 with HCN2 have a reduced cAMP sensitivity. This observation might in part contribute to the complex changes in the cAMP-sensitivity of *I*_h_ which was observed in TC neurons of the WAG/Rij strain (Kanyshkova et al., 2012). (4) While WAG-HCN1 was not detected at P7, the WAG-HCN1 variant was up-regulated at postnatal ages P30 and P90. These findings indicate that the novel HCN1 channel variant appears in WAG/Rij rats already at an age period where SWD are not yet present (van Luijtelaar and Sitnikova, 2006), but the fate of the future epileptic phenotype is critically determined by HCN expression levels (Schridde et al., 2006; Blumenfeld et al., 2008). Most importantly, the increased *I*_h_ current density in WAG/Rij strains between P15 and P30, peaking at P90 is not caused by an enhanced HCN1 transcription alone, but also by the appearance of the novel WAG-HCN1 isoform in this time window.

The conclusions discussed above are limited by the fact that the expression of the WAG-HCN1 variant in the thalamus was only determined on mRNA level in the present study. We tried to overcome this limitation by generating four monoclonal antibodies directed against overlapping fragments of a short peptide containing the WAG-HCN1 polyserine motif (SNSSSSSSSKVD; Abmart, Inc., Shanghai, China). However, none of these antibodies was able to detect WAG-HCN1 protein isolated from COS7 cells transfected with EGFP-tagged WAG-HCN1 channels in Western blots (data not shown). Therefore, the relative expression of WAG-HCN1 channels in the thalamus on protein level currently remains unresolved.

Another open question is the mechanism of how WAG-HCN1 transcripts are generated in a tissue- and species-dependent manner. The WAG-HCN1 is not generated by a classical alternative splicing, as it lacks parts of the coding region in the middle of the first exon. However, as the genomic sequence of WAG and ACI strains are identical, our data support a scenario in WAG/Rij rats where not yet identified mechanisms lead to post-transcriptional modifications of HCN1 and the generation of WAG-HCN1.

It is known that HCN1, HCN2, and HCN4 channels remain functional even after deletion of the CNBD or the entire C-terminus (Wainger et al., 2001; Schulze-Bahr et al., 2003). However, so far most of the data published indicates that alteration or deletion of C-terminal portions of HCN channels cause a decreased current expression and not a current increase. However, our observation that the deletion of the proximal part of the HCN1 N-terminus results in a current increase, is supported by two studies in which an N-terminal deletion in HCN1 leads to an increased current amplitude or surface expression (Vemana et al., 2008; Pan et al., 2015). Vemana et al. (2008) state: “*The HCN1 channels had the COOH terminus deleted and a small region in the NH_2_ terminus deleted that maximize the expression in oocytes.*” A recent study describes a N-terminal ER export signal (20 amino acids) essential for the targeting of HCN1 to the plasma membrane of the inner segment of *Xenopus* photoreceptors (Pan et al., 2015). However, this motif is not affected in WAG-HCN1, as it is located shortly after the deletion, starting at amino acid 75. Most importantly within this study the localization of a series of N-terminal truncation mutants of GFP-HCN1 were analyzed. Here it can be noted that a WAG-like deletion of the first 55 amino acids of HCN1 results in a marked increase of fluorescence at the surface membrane (compare WT in Figure 2A versus 3B in Pan et al., 2015). On the other hand the polyserine motif in WAG-HCN1 channels appears to be of minor modulatory relevance, as we did not observe an altered protein kinase regulation of the WAG-HCN1 channel. However, the N-terminal deletion of the 37 amino acids alone cannot reproduce the electrophysiological phenotype of the WAG-HCN1 channels. We found that the loss of the GNSVCF wild-type sequence, directly following the 37 amino acid deletion, must contribute to the gain-of-function. This could be based on at least two possible mechanisms. First, a change in protein sequence could result in structural changes. Second, a potential motif that retains HCN1 is deleted. Third, the altered sequence might impair an interaction with a protein binding to the N-terminus of the channel. A possible binding site might include a part of the deleted sequence plus the GNSVCF motif. The WAG-HCN1 might have an impaired interaction with a protein involved in the channel trafficking, a structural protein or a channel subunit. HCN1 channels have a proline-rich N-terminus which is a putative interaction site for SH2-, SH3-, or WW-domains (Kay et al., 2000). In addition, HCN1 channels are known to interact with TRIP8b and Filamin A (Gravante et al., 2004; Santoro et al., 2004). Filamin A causes a negative shift in the voltage dependence of activation and causes reduction in current amplitude (Gravante et al., 2004). Thus, the loss of interaction with Filamin A could be a possible mechanism for the current increase seen for WAG-HCN1. However, it would not explain the negative shift in the voltage dependence observed for heteromeric channels of WAG-HCN1 with HCN2. TRIP8b also affects HCN1 current amplitudes, but this effect is dependent on the isoform of TRIP8b (Santoro et al., 2004; Lewis et al., 2009; Santoro et al., 2009; Zolles et al., 2009). Also with TRIP8b, the voltage dependence of HCN1 is shifted to more hyperpolarized potentials (Lewis et al., 2009; Santoro et al., 2009). Thus, also a loss of interaction with TRIP8b cannot explain the effects observed with WAG-HCN1. In addition, both proteins, Filamin A and TRIP8b interact with the C-terminus of HCN1, making a loss-of-interaction with these proteins an unlikely mechanism. Although one cannot completely rule out that strong changes in the N-terminal sequence result in an altered stabilization of a channel tetramer and their respective interaction partners. Whether the effects observed with WAG-HCN1 result from a change in interaction with a known or unknown protein partner or whether the effects are based on structural changes in the WAG-HCN1 constructs remains to be elucidated in future studies. By using short deletions in HCN1 it may be possible to elucidate the exact region which is gaining in the surface expression of the channel and subsequently, the augmentation of *I*_h_.

The identification of a WAG-HCN1 variant with a specific gain-of-function and an impact on the biophysical properties of heteromeric HCN channels raises many new questions and prompts new biophysical studies in neuronal tissues of the WAG/Rij strain. Future studies might focus for example on the tissue specific expression of the WAG-HCN1 variant and whether this isoform also underlies a specific ontogenetic transcriptional regulation. We conclude that the altered functional properties of the WAG-HCN1 variant and the heteromeric channels with WAG-HCN1 may help to explain many previous observations in native tissue of the WAG/Rij strain. In addition, the presence of the novel WAG-HCN1 variant might represent one of several factors for the epileptic phenotype of the WAG/Rij strain.

## Author Contributions

KW performed all TEVC recordings. TK cloned the HCN1 constructs and performed Western blot and PCR experiments. SR constructed different HCN channel constructs. KW and MN performed the fluorescence microscopy. NS and AKK performed patch clamp and TEVC recordings and Western blot experiments. JF-O and SB performed Western blot experiments. Data analyses were done by KW, TK, NS, AKK, JF-O, SB, TB, and ND. ND, TB, and SGM designed the study. KW, ND, and TB wrote the first draft of the manuscript. All authors edited and commented on the manuscript.

## Conflict of Interest Statement

The authors declare that the research was conducted in the absence of any commercial or financial relationships that could be construed as a potential conflict of interest.

## Abbreviations

ACI: August Copenhagen Irish rat
dLGN: dorsal part of the lateral geniculate nucleus
HCN: hyperpolarization-activated cyclic nucleotide-gated cation channel
*I*_h_: hyperpolarization-activated inward current
P: postnatal day
SSC: somatosensory cortex
SWD: spike-and-wave discharge
TC neuron: thalamocortical relay neuron
TEVC: two-electrode voltage clamp
TRIP8b: tetratricopeptide repeat-containing Rab8b-interacting protein
WAG/Rij: Wistar Albino Glaxo rats from Rijswijk

## References

[B1] Akiyama T. Ishida J. Nakagawa S. Ogawara H. Watanabe S. Itoh N. (1987). Genistein, a specific inhibitor of tyrosine-specific protein kinases. *J. Biol. Chem.* 262 5592–5595. 3106339

[B2] Biel M. Wahl-Schott C. Michalakis S. Zong X. (2009). Hyperpolarization-activated cation channels: from genes to function. *Physiol. Rev.* 89 847–885. 10.1152/physrev.00029.2008 19584315

[B3] Blumenfeld H. Klein J. P. Schridde U. Vestal M. Rice T. Khera D. S. (2008). Early treatment suppresses the development of spike-wave epilepsy in a rat model. *Epilepsia* 49 400–409. 10.1111/j.1528-1167.2007.01458.x 18070091 PMC3143182

[B4] Budde T. Caputi L. Kanyshkova T. Staak R. Abrahamczik C. Munsch T. (2005). Impaired regulation of thalamic pacemaker channels through an imbalance of subunit expression in absence epilepsy. *J. Neurosci.* 25 9871–9882. 10.1523/JNEUROSCI.2590-05.2005 16251434 PMC6725576

[B5] Cain S. M. Tyson J. R. Jones K. L. Snutch T. P. (2014). Thalamocortical neurons display suppressed burst-firing due to an enhanced I_h_ current in a genetic model of absence epilepsy. *Pflügers Arch.* 467 1367–1382. 10.1007/s00424-014-1549-4 24953239 PMC4435665

[B6] Chen S. Wang J. Siegelbaum S. A. (2001). Properties of hyperpolarization-activated pacemaker current defined by coassembly of HCN1 and HCN2 subunits and basal modulation by cyclic nucleotide. *J. Gen. Physiol.* 117 491–504. 10.1085/jgp.117.5.491 11331358 PMC2233656

[B7] Chijiwa T. Mishima A. Hagiwara M. Sano M. Hayashi K. Inoue T. (1990). Inhibition of forskolin-induced neurite outgrowth and protein phosphorylation by a newly synthesized selective inhibitor of cyclic AMP-dependent protein kinase, N-[2-(p-bromocinnamylamino)ethyl]-5-isoquinolinesulfonamide (H-89), of PC12D pheochromocytoma cells. *J. Biol. Chem.* 265 5267–5272. 2156866

[B8] Chow S. S. Van Petegem F. Accili E. A. (2012). Energetics of cyclic AMP binding to HCN channel C terminus reveal negative cooperativity. *J. Biol. Chem.* 287 600–606. 10.1074/jbc.M111.269563 22084239 PMC3249114

[B9] Chung W. K. Shin M. Jaramillo T. C. Leibel R. L. Leduc C. A. Fischer S. G. (2009). Absence epilepsy in apathetic, a spontaneous mutant mouse lacking the h channel subunit, HCN2. *Neurobiol. Dis.* 33 499–508. 10.1016/j.nbd.2008.12.004 19150498 PMC2643333

[B10] Coenen A. M. van Luijtelaar E. L. (2003). Genetic animal models for absence epilepsy: a review of the WAG/Rij strain of rats. *Behav. Genet.* 33 635–655. 10.1023/A:1026179013847 14574120

[B11] Couldwell W. T. Hinton D. R. He S. Chen T. C. Sebat I. Weiss M. H. (1994). Protein kinase C inhibitors induce apoptosis in human malignant glioma cell lines. *FEBS Lett.* 345 43–46. 10.1016/0014-5793(94)00415-3 8194597

[B12] Crunelli V. Leresche N. (2002). Childhood absence epilepsy: genes, channels, neurons and networks. *Nat. Rev. Neurosci.* 3 371–382. 10.1038/nrn811 11988776

[B13] Davis P. D. Hill C. H. Keech E. Lawton G. Nixon J. S. Sedgwick A. D. (1989). Potent selective inhibitors of protein kinase C. *FEBS Lett.* 259 61–63. 10.1016/0014-5793(89)81494-2 2532156

[B14] Depaulis A. van Luijtelaar G. (2006). “Genetic models of absence epilepsy in the rat,” in *Models of Seizures and Epilepsy* eds Pitk änen A. Schwartzkroin P. A. Moshé S. L. (Amsterdam: Elsevier Academic Press) 233–248.

[B15] DiFrancesco J. C. DiFrancesco D. (2015). Dysfunctional HCN ion channels in neurological diseases. *Front. Cell. Neurosci.* 9:71 10.3389/fncel.2015.00071 PMC4354400 25805968

[B16] Di Pasquale E. Keegan K. D. Noebels J. L. (1997). Increased excitability and inward rectification in layer V cortical pyramidal neurons in the epileptic mutant mouse Stargazer. *J. Neurophysiol.* 77 621–631. 9065835 10.1152/jn.1997.77.2.621

[B17] Gravante B. Barbuti A. Milanesi R. Zappi I. Viscomi C. Difrancesco D. (2004). Interaction of the pacemaker channel HCN1 with filamin A. *J. Biol. Chem.* 279 43847–43853. 10.1074/jbc.M401598200 15292205

[B18] Huguenard J. R. McCormick D. A. (2007). Thalamic synchrony and dynamic regulation of global forebrain oscillations. *Trends Neurosci.* 30 350–356. 10.1016/j.tins.2007.05.007 17544519

[B19] Kanyshkova T. Meuth P. Bista P. Liu Z. Ehling P. Caputi L. (2012). Differential regulation of HCN channel isoform expression in thalamic neurons of epileptic and non-epileptic rat strains. *Neurobiol. Dis.* 45 450–461. 10.1016/j.nbd.2011.08.032 21945537 PMC3225716

[B20] Kanyshkova T. Pawlowski M. Meuth P. Dube C. Bender R. A. Brewster A. L. (2009). Postnatal expression pattern of HCN channel isoforms in thalamic neurons: relationship to maturation of thalamocortical oscillations. *J. Neurosci.* 29 8847–8857. 10.1523/JNEUROSCI.0689-09.2009 19587292 PMC2768285

[B21] Kay B. K. Williamson M. P. Sudol M. (2000). The importance of being proline: the interaction of proline-rich motifs in signaling proteins with their cognate domains. *FASEB J.* 14 231–241. 10657980

[B22] Kole M. H. Brauer A. U. Stuart G. J. (2007). Inherited cortical HCN1 channel loss amplifies dendritic calcium electrogenesis and burst firing in a rat absence epilepsy model. *J. Physiol.* 578 507–525. 10.1113/jphysiol.2006.122028 17095562 PMC2075144

[B23] Kuisle M. Wanaverbecq N. Brewster A. L. Frere S. G. Pinault D. Baram T. Z. (2006). Functional stabilization of weakened thalamic pacemaker channel regulation in rat absence epilepsy. *J. Physiol.* 575 83–100. 10.1113/jphysiol.2006.110486 16728450 PMC1819420

[B24] Leresche N. Lambert R. C. Errington A. C. Crunelli V. (2012). From sleep spindles of natural sleep to spike and wave discharges of typical absence seizures: is the hypothesis still valid? *Pflügers Arch.* 463 201–212. 10.1007/s00424-011-1009-3 21861061 PMC3256322

[B25] Lewis A. S. Schwartz E. Chan C. S. Noam Y. Shin M. Wadman W. J. (2009). Alternatively spliced isoforms of TRIP8b differentially control h channel trafficking and function. *J. Neurosci.* 29 6250–6265. 10.1523/JNEUROSCI.0856-09.2009 19439603 PMC2730639

[B26] Lolicato M. Nardini M. Gazzarrini S. Moller S. Bertinetti D. Herberg F. W. (2011). Tetramerization dynamics of C-terminal domain underlies isoform-specific cAMP gating in hyperpolarization-activated cyclic nucleotide-gated channels. *J. Biol. Chem.* 286 44811–44820. 10.1074/jbc.M111.297606 22006928 PMC3247997

[B27] Ludwig A. Budde T. Stieber J. Moosmang S. Wahl C. Holthoff K. (2003). Absence epilepsy and sinus dysrhythmia in mice lacking the pacemaker channel HCN2. *EMBO J.* 22 216–224. 10.1093/emboj/cdg032 12514127 PMC140107

[B28] Lüttjohann A. van Luijtelaar G. (2015). Dynamics of networks during absence seizure’s on– and offset in rodents and man. *Front. Physiol.* 6:16 10.3389/fphys.2015.00016 PMC4318340 25698972

[B29] Much B. Wahl-Schott C. Zong X. Schneider A. Baumann L. Moosmang S. (2003). Role of subunit heteromerization and N-linked glycosylation in the formation of functional hyperpolarization-activated cyclic nucleotide-gated channels. *J. Biol. Chem.* 278 43781–43786. 10.1074/jbc.M306958200 12928435

[B30] Pan Y. Laird J. G. Yamaguchi D. M. Baker S. A. (2015). An N-terminal ER export signal facilitates the plasma membrane targeting of HCN1 channels in photoreceptors. *Invest. Ophthalmol. Vis. Sci.* 56 3514–3521. 10.1167/ioversus15-16902 26030105 PMC4464044

[B31] Pinault D. O’Brien T. J. (2005). Cellular and network mechanisms of genetically-determined absence seizures. *Thalamus Relat. Syst.* 3 181–203. 10.1017/S1472928807000209 21909233 PMC3168114

[B32] Ruegg U. T. Burgess G. M. (1989). Staurosporine, K-252 and UCN-01: potent but nonspecific inhibitors of protein kinases. *Trends Pharmacol. Sci.* 10 218–220. 2672462 10.1016/0165-6147(89)90263-0

[B33] Santoro B. Piskorowski R. A. Pian P. Hu L. Liu H. Siegelbaum S. A. (2009). TRIP8b splice variants form a family of auxiliary subunits that regulate gating and trafficking of HCN channels in the brain. *Neuron* 62 802–813. 10.1016/j.neuron.2009.05.009 19555649 PMC2720631

[B34] Santoro B. Wainger B. J. Siegelbaum S. A. (2004). Regulation of HCN channel surface expression by a novel C-terminal protein-protein interaction. *J. Neurosci.* 24 10750–10762. 10.1523/JNEUROSCI.3300-04.2004 15564593 PMC6730122

[B35] Schridde U. Strauss U. Brauer A. U. van Luijtelaar G. (2006). Environmental manipulations early in development alter seizure activity, Ih and HCN1 protein expression later in life. *Eur. J. Neurosci.* 23 3346–3358. 10.1111/j.1460-9568.2006.04865.x 16820024

[B36] Schulze-Bahr E. Neu A. Friederich P. Kaupp U. B. Breithardt G. Pongs O. (2003). Pacemaker channel dysfunction in a patient with sinus node disease. *J. Clin. Invest.* 111 1537–1545. 10.1172/JCI16387 12750403 PMC155041

[B37] Strauss U. Kole M. H. Brauer A. U. Pahnke J. Bajorat R. Rolfs A. (2004). An impaired neocortical Ih is associated with enhanced excitability and absence epilepsy. *Eur. J. Neurosci.* 19 3048–3058. 10.1111/j.0953-816X.2004.03392.x 15182313

[B38] Streit A. K. Netter M. F. Kempf F. Walecki M. Rinné S. Bollepalli M. K. (2011). A specific two-pore domain potassium channel blocker defines the structure of the TASK-1 open pore. *J. Biol. Chem.* 286 13977–13984. 10.1074/jbc.M111.227884 21362619 PMC3077598

[B39] van Luijtelaar G. Hramov A. Sitnikova E. Koronovskii A. (2011a). Spike-wave discharges in WAG/Rij rats are preceded by delta and theta precursor activity in cortex and thalamus. *Clin. Neurophysiol.* 122 687–695. 10.1016/j.clinph.2010.10.038 21093357

[B40] van Luijtelaar G. Sitnikova E. Littjohann A. (2011b). On the origin and suddenness of absences in genetic absence models. *Clin. EEG Neurosci.* 42 83–97. 10.1177/155005941104200209 21675598

[B41] van Luijtelaar G. Sitnikova E. (2006). Global and focal aspects of absence epilepsy: the contribution of genetic models. *Neurosci. Biobehav. Rev.* 30 983–1003. 10.1016/j.neubiorev.2006.03.002 16725200

[B42] van Luijtelaar G. Zobeiri M. (2014). Progress and outlooks in a genetic absence epilepsy model (WAG/Rij). *Curr. Med. Chem.* 21 704–721. 10.2174/0929867320666131119152913 24251564

[B43] Vemana S. Pandey S. Larsson H. P. (2008). Intracellular Mg2+ is a voltage-dependent pore blocker of HCN channels. *Am. J. Physiol. Cell Physiol.* 295 C557–C565. 10.1152/ajpcell.00154.2008 18579800 PMC2518427

[B44] Wainger B. J. Degennaro M. Santoro B. Siegelbaum S. A. Tibbs G. R. (2001). Molecular mechanism of cAMP modulation of HCN pacemaker channels. *Nature* 411 805–810. 10.1038/35081088 11459060

[B45] Yue T. L. Wang C. Romanic A. M. Kikly K. Keller P. Dewolf W. E. (1998). Staurosporine-induced apoptosis in cardiomyocytes: a potential role of caspase-3. *J. Mol. Cell. Cardiol.* 30 495–507. 10.1006/jmcc.1997.0614 9515027

[B46] Zolles G. Wenzel D. Bildl W. Schulte U. Hofmann A. Muller C. S. (2009). Association with the auxiliary subunit PEX5R/Trip8b controls responsiveness of HCN channels to cAMP and adrenergic stimulation. *Neuron* 62 814–825. 10.1016/j.neuron.2009.05.008 19555650

